# Streptococcal IdeS: therapeutic potential for Guillain–Barré syndrome

**DOI:** 10.1038/srep10809

**Published:** 2015-07-21

**Authors:** Ryo Takahashi, Nobuhiro Yuki

**Affiliations:** 1 Department of Medicine, Yong Loo Lin School of Medicine, National University of Singapore, Singapore; 2 Physiology, Yong Loo Lin School of Medicine, National University of Singapore, Singapore

## Abstract

Plasma exchange and intravenous immunoglobulin are effective in treating Guillain–Barré syndrome (GBS) probably because the former removes IgG autoantibodies and complement and the latter inhibits complement activation subsequent to the autoantibody binding to peripheral nerve antigens. IgG degrading enzyme of *Streptococcus pyogenes* (IdeS) can cleave the pathogenic autoantibodies into F(ab’)_2_ and Fc. The purpose of this study is to show whether IdeS has novel therapeutic potential for GBS. Sera with anti-ganglioside IgG antibodies from 15 patients with GBS or Miller Fisher syndrome were used. We tested whether IdeS cleaved the anti-ganglioside IgG antibodies and inhibited deposition of activated complement component on ELISA plates. IdeS efficiently cleaved IgG and blocked complement activation mediated by anti-GM1, anti-GD1a and anti-GQ1b IgG antibodies. IdeS has therapeutic potential for GBS and related conditions.

Guillain–Barré syndrome (GBS) is pathologically divided into two major forms, acute inflammatory demyelinating polyneuropathy (AIDP) and acute motor axonal neuropathy (AMAN)^1^. Ganglioside mimicry of micro-organisms can induce anti-GM1 or anti-GD1a IgG antibodies in patients, and the autoantibodies bind to GM1 or GD1a at the nodes of Ranvier in the spinal anterior roots^2^,^3^. The binding activates complement *in situ*, leading to the disappearance of voltage-gated sodium channel clusters and disruption of axo-glial junction^3^,^4^. These result in motor nerve conduction failure and muscle weakness. Although pathogenic antibodies have yet to be determined in AIDP, autopsy studies showed that complement activation at the outer surface of Schwann cells is probably subsequent to IgG antibodies binding to targets on Schwann cells^5^. Plasma exchange and intravenous immunoglobulin (IVIG) are effective for treating GBS most likely due to the fact that plasma exchange removes IgG and complement, and IVIG can neutralize pathogenic IgG autoantibodies and inhibit complement activation^6^,^7^. However, even when patients are treated with either immunotherapy, 20% remain severely disabled and 5% die^8^. More effective treatments are required based on the understanding of the immunopathogenesis.

Immunoglobulin G-degrading enzyme of *Streptococcus pyogenes* (IdeS), which cleaves IgG antibodies into F(ab’)_2_ and Fc fragments, is secreted by *S. pyogenes*^9^. IdeS is one of the virulence factors for the bacterium, which helps it to escape from the host’s immunological defenses, such as phagocytosis and complement activation, by removing the Fc region from IgG targeting, e.g. bacterial surface antigens. By taking advantage of this action, IdeS has been shown to have therapeutic effects in several autoimmune disease models mediated by pathogenic autoantibodies^10^. IdeS seems to be a promising treatment because the cleavage of IgG antibodies inhibits complement activation subsequent to the formation of the immune-complex. Here we demonstrate that IdeS blocked complement activation mediated by anti-ganglioside IgG antibodies *in vitro*.

## Results

### IdeS efficiently cleaved IgG and blocked complement activation mediated by anti-GM1, anti-GD1a and anti-GQ1b IgG antibodies

**Figure 1:**
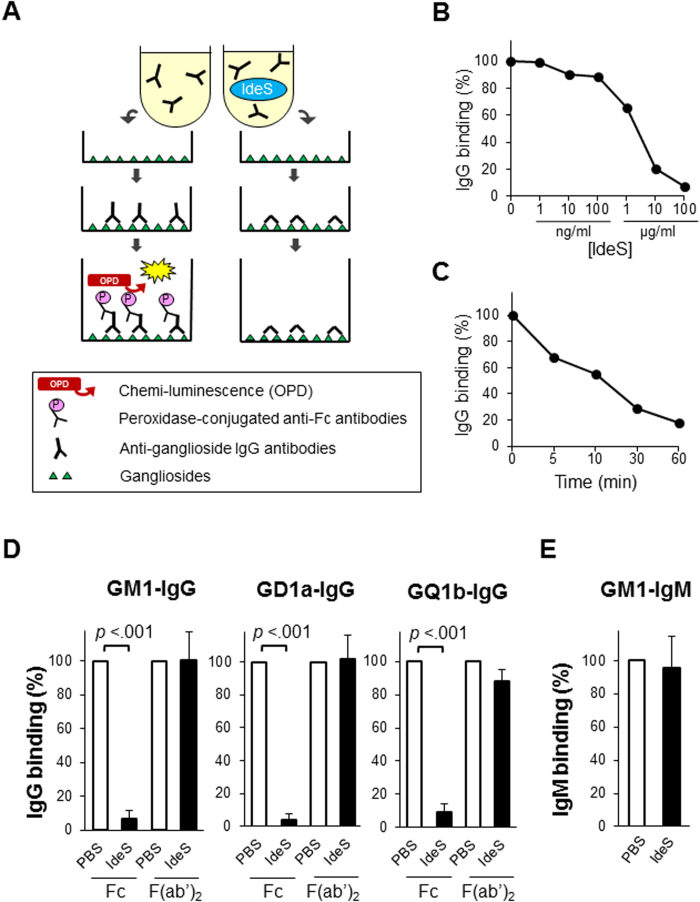
(**A**) Schema of IdeS treatment for binding of autoantibodies. Anti-ganglioside antibodies in the diluted patients’ sera bind to the ganglioside coating on the microtiter plates. IdeS cleaves IgG antibodies into F(ab’)_2_ and Fc fragments. Peroxidase-conjugated anti-human IgG (Fc) does not bind to the F(ab’)_2_ residue and does not work as the luminescent substrate in IdeS treated plates. (**B**) Concentration dependence of IdeS treatment. The representative change of GM1-IgG binding depends on its concentration. The clearance of IgG (Fc) accumulated to the highest levels at a concentration of 10 μg/ml or more of IdeS. (**C**) Time to react after adding IdeS. The binding of GM1-IgG halved after around 10 minutes and was almost eliminated after one hour. (**D**) The binding of IgG was detected by ELISA with both anti-Fc and anti-F(ab’)_2_ antibodies. Fc deposition was degraded by IdeS (10 μg/ml), whereas F(ab’)_2_ deposition remained unaltered. IdeS inhibited the Fc deposition of not only anti-GM1 IgG (n = 5) but also anti-GD1a IgG (n = 5) and anti-GQ1b IgG (n = 5) antibodies. (**E**) The binding of IgM was detected by ELISA. IdeS did not affect the binding of anti-GM1 IgM antibodies (n = 5).

**Figure 2:**
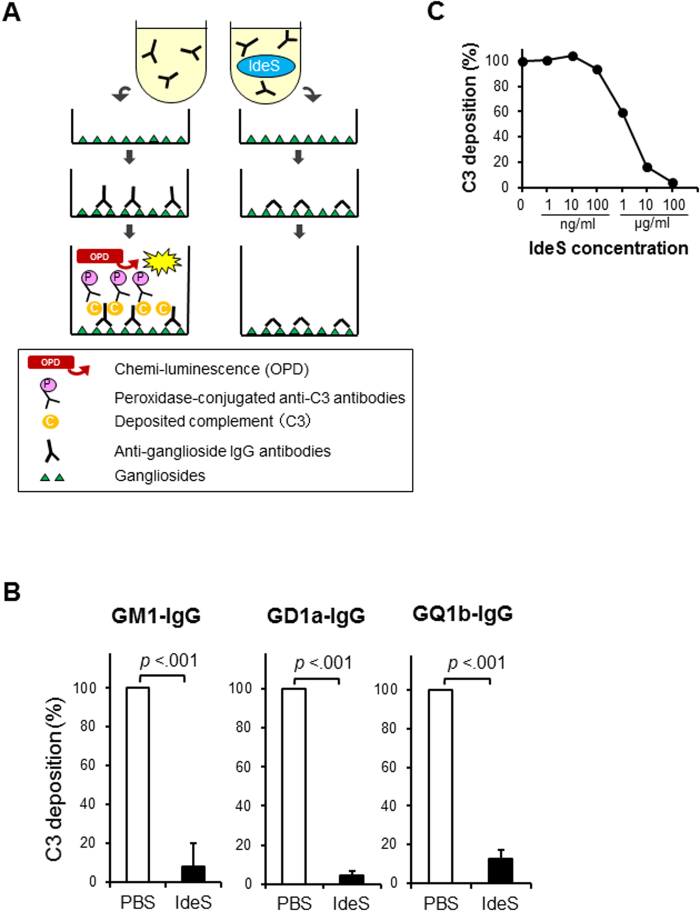
(**A**) Schema of IdeS treatment for complement deposition. Complement C3 deposition mediated by anti-ganglioside IgG antibodies was detected on the microtiter plates. IdeS treatment cleaved IgG autoantibodies and prevented subsequent complement activation. (**B**) Blocking effect of IdeS on complement deposition. IdeS blocked C3 deposition mediated by IgG autoantibodies against GM1, GD1a and GQ1b, respectively. (**C**) Concentration dependence of IdeS treatment. The representative change of C3 deposition mediated by GM1-IgG depends on its concentration. The blocking effect of C3 accumulated to the highest levels at a concentration of 10 μg/ml or more of IdeS.

The established assays demonstrated the binding of autoantibodies to each ganglioside and the deposition of active complement component. The detection of Fc domain was obscured due to cleavage by IdeS and subsequent rinsing out. Nevertheless F(ab’)_2_ remained to bind stably to the ganglioside coating on the microtiter plates (Fig. 1A and 2A). The clearance of Fc depended on the concentration of IdeS (Fig. 1B) as well as the time after the addition of IdeS to the serum (Fig. 1C). The cleaving effect emerged in a few minutes and reached the maximum in one hour. IdeS cleaved all the anti-GM1, anti-GD1a and anti-GQ1b IgG antibodies (Fig. 1D). Fc deposition was degraded by IdeS (10 μg/ml), whereas F(ab’)_2_ deposition remained unaltered. Thus, the subsequent complement deposition mediated by anti-ganglioside IgG autoantibodies was inhibited by IdeS, resulting in the blocking of the C3 deposition (Fig. 2B). The blocking effect depended on the concentration of IdeS as well as the clearance of Fc (Fig. 2C). In contrast, IdeS did not affect the binding of anti-GM1 IgM antibodies (Fig. 1E).

## Discussion

Anti-ganglioside antibodies binding followed by complement activation causes nerve injury in the axonal form of GBS^2^,^3^,^4^. Removal or scavenging of pathological autoantibodies is thought to be one of the most important mechanisms of plasma exchange and IVIG^6^,^11^. Complement inhibitors such as eculizumab and nafamostat mesilate prevented nerve injury in animal models of GBS^12^,^13^. Therefore, therapeutic approaches to decreasing both pathological antibodies and the subsequent deposition of complement by immunotherapies are considered to effectively ameliorate the severity and outcome of GBS in patients.

In the current study, we showed that IdeS successfully cleaved the pathological antibodies and blocked the activation of complement. It is considered that F(ab’)_2_ domain of autoantibodies binding to ganglioside cannot cause pathogenesis independently in GBS because it lacks Fc domain which binds and activates immune effectors. This suggests that IdeS could suppress the activation of complement on the axolemma of motor fibers at the nodes of Ranvier where pathological anti-ganglioside autoantibodies deposit and thereby prevent nerve injury. We confirmed that IdeS cleaved the Fc domains similarly both before and after anti-ganglioside antibodies bind to ganglioside (data not shown). This suggests that IdeS could cleave circulating anti-ganglioside IgG antibodies before their binding to the antigen and remove the Fc domains of the immune complexes binding to the nerve antigens.

Animal experiments have shown that IdeS treatment is strong enough to prevent or cure the diseases induced by autoantibodies. IdeS treatment of mice with collagen-antibody-induced arthritis reduced the severity of the arthritis when administered within 24 hours of the onset of clinical arthritis and prevented an antibody-induced relapse^14^. In a mouse model of immune thrombocytopenic purpura with IgG antibodies against platelet surface antigens, profoundly thrombocytopenic animals were treated and cured by a single injection of IdeS^15^. Severe albuminuria in mice induced by anti-glomerular basement membrane antibodies was completely prevented by IdeS, accompanied by a significant reduction of the deposition of complement components^16^. In a mouse model of neuromyelitis optica induced by anti-aquaporin-4 antibodies, IdeS treatment greatly reduced pathological lesions^17^. These reports showed that IdeS reduced the pathogenesis caused by antibody-mediated complement-dependent cytotoxicity and antibody-dependent cell-mediated cytotoxicity. Other autoimmune diseases with inflammation induced by pathological autoantibodies could be possible candidates for IdeS treatment.

Anti-IdeS antibodies may be present in humans and may increase in number after therapeutic use of IdeS because IdeS is a heterologous protein secreted by *S. pyogenes*. Streptokinase, another enzyme secreted by several streptococci has been used as an effective and safe thrombolysis medication by activating plasminogen^18^. Repeated use of IdeS could increase the number of anti-IdeS IgG antibodies, which might neutralize and suppress the effect of IdeS and produce anti-IdeS antibodies, which may cause severe adverse reactions such as hypersensitivity. Therefore, single rather than repeated use of IdeS is advised. From this viewpoint, GBS is one of the most promising candidates for IdeS treatment because GBS is an acute and monophasic disease. Furthermore, IdeS may increase risk of infection in GBS patients because of its characteristic as a virulence factor for microbes. There is another concern that naturally occurring, anti-hinge antibodies may generate complement C3b_2_-containing immune complexes which could stimulate complement amplification^19^. However, a phase I clinical trial in healthy volunteers was safely executed^20^. Based on those results, subjects have been recruited for a phase II trial designed to evaluate the safety and efficacy of IdeS in patients with chronic kidney diseases who are scheduled for kidney transplantation with donor-specific HLA antibodies because they require plasma exchange before the transplantation^21^.

Clinical trials performed in Europe and North America showed plasma exchange is effective for GBS^1^, suggesting that IgG autoantibodies play a crucial role for the development of AIDP, a predominant form of GBS in western countries. IdeS has been developed for patients who require plasma exchange. We believe that IdeS is effective for demyelinating GBS as well, and we are also planning a phase II trial in patients with GBS.

In conclusion, we found that IdeS cleaved the pathological anti-ganglioside antibodies and blocked the subsequent complement activity mediated by autoantibodies. These results suggest that IdeS is an effective treatment for GBS.

## Methods

### Serum samples

The study included patients with GBS, Miller Fisher syndrome and multifocal motor neuropathy, admitted to Dokkyo Medical University (Tochigi, Japan) who fulfilled the published diagnostic criteria^22^,^23^. Serum samples were chosen from the patients with GBS associated with anti-GM1 (n = 5) or anti-GD1a IgG antibodies (n = 5); Miller Fisher syndrome associated with anti-GQ1b IgG antibodies (n = 5); or multifocal motor neuropathy associated with anti-GM1 IgM antibodies (n = 5), which had been kept at −80 °C before use. All experiments were performed in accordance with NMEC Ethical Guidelines on Research Involving Human Subjects^24^. Written informed consent was obtained from every patient. Normal human sera were obtained from five healthy subjects as a source of complement. The study was approved by the Dokkyo Medical University Ethics Committee and National University Singapore Medical Research Ethics Committee.

### ELISA

Phosphate-buffered saline (PBS) containing 0.5% casein sodium salt was used for each dilution. The serum dilutions were titrated so that the optical density values showed from 2.0 to 3.0 with anti-ganglioside antibody-positive sera. Sera from patients with anti-GM1, anti-GD1a and anti-GQ1b IgG as well as anti-GM1 IgM antibodies were diluted at 1:500 and added to respective ganglioside-coated plates. The microtiter plates were incubated for one hour at 37 °C. To detect IgG binding, peroxidase-conjugated goat anti-human IgG antibodies recognizing the Fc (1:2,000; Sigma-Aldrich, Dorset, UK) or F(ab’)_2_ domain (1:2,000; Thermo Scientific, Rockford, IL) were used. To detect IgM binding, peroxidase-conjugated rabbit anti-human IgM antibodies (1:2,000; Sigma-Aldrich) were used after washing with PBS containing 0.05% Tween 20 (PBS-T). A complement deposition assay was performed in quintuplicate as described previously with minor modification^25^. Normal human sera diluted at 1:100 were added as a source of complement. After incubation for one hour at 37 °C and subsequent washing with PBS-T, peroxidase-conjugated goat anti-human C3 antibodies (1:2,000; MP Biomedicals LLC, Solon, OH, USA) were added. After further incubation and washing with PBS-T, each plate was developed.

### IdeS treatment

IdeS was kindly provided by Hansa Medical AB (Lund, Sweden). Diluted IdeS in 50 μl of PBS was used in different concentrations from 1 ng/ml to 100 μg/ml in individual wells of the ELISA plate to generate a dose-response curve. To investigate whether IdeS cleaves Fc domain of anti-ganglioside antibodies and blocks the deposition of complement mediated by anti-ganglioside antibodies, patients’ sera mixed with IdeS were incubated on microtiter plates for 1 hour at 37 °C. The process is represented schematically in Fig. 1A and 2A. The optimal blocking dose of IdeS was determined to be 10 μg/ml and this dose was used in the sequential assays. PBS, which did not contain IdeS, was used as a control and the relative IgG binding rates and the C3 deposition rates were evaluated respectively.

### Statistics

Data were analyzed using JMP statistical discovery software version 10.0.2 (SAS Institute Inc., Cary, NC). Statistical significance was evaluated using the Mann–Whitney *U*-test. Differences where *P* < 0.05 were considered significant.

## Additional Information

**How to cite this article**: Takahashi, R. and Yuki, N. Streptococcal IdeS: therapeutic potential for Guillain-Barré syndrome. *Sci. Rep.* **5**, 10809; doi: 10.1038/srep10809 (2015).
